# Bazedoxifene Attenuates Abdominal Aortic Aneurysm Formation *via* Downregulation of Interleukin-6/Glycoprotein 130/Signal Transducer and Activator of Transcription 3 Signaling Pathway in Apolipoprotein E–Knockout Mice

**DOI:** 10.3389/fphar.2020.00392

**Published:** 2020-04-17

**Authors:** Dan Yan, Haiyan Ma, Wei Shi, Pengcheng Luo, Tianshu Liu, Junyi Guo, Maocai Zhai, Jingwen Tao, Shengqi Huo, Chenglong Li, Jiayuh Lin, Sheng Li, Jiagao Lv, Cuntai Zhang, Li Lin

**Affiliations:** ^1^ Division of Cardiology, Department of Internal Medicine, Tongji Hospital, Tongji Medical College, Huazhong University of Science and Technology, Wuhan, China; ^2^ Department of Geriatrics, Tongji Hospital, Tongji Medical College, Huazhong University of Science and Technology, Wuhan, China; ^3^ Division of Cardiology, Department of Internal Medicine, First People’s Hospital of Shangqiu, Shangqiu, China; ^4^ Department of Medicinal Chemistry, College of Pharmacy, University of Florida, Gainesville, FL, United States; ^5^ Department of Biochemistry and Molecular Biology, University of Maryland School of Medicine, Baltimore, MD, United States

**Keywords:** abdominal aortic aneurysm, inflammation, bazedoxifene, IL-6, STAT3

## Abstract

Abdominal aortic aneurysm (AAA) is a chronic inflammatory disease characterized by aortic dilatation and predominantly affects an elderly population. Accumulating evidence suggests that Interleukin-6 (IL-6) and the signal transducer and activator of transcription 3 (STAT3) play an important role in formation of AAAs. However, it remains unclear whether Bazedoxifene (BAZ) could suppress the activation of IL-6/GP130/STAT3 in vascular cells and the formation of AAA. Here we explored the effect of BAZ on AngII-stimulated AAA formation. ApoE^–/–^ mice infused with AngII for 28 days using osmotic minipumps were treated with placebo or 5mg/kg BAZ. In our results most of the AngII-induced mice developed AAA with exacerbated inflammation, degradation of elastin fibers, STAT3 phosphorylation, and increased expression of matrix metalloproteinases (MMPs). These effects were markedly attenuated by BAZ. Furthermore, BAZ suppressed the stimuli-induced (IL-6 or AngII) expression of P-STAT3, MMP2 and MMP9 in vascular smooth muscle cells (VSMCs). BAZ inhibited wound healing, colony formation and suppressed STAT3 nuclear translocation *in vitro*. In conclusion, these results indicated that BAZ downregulated IL-6/GP130/STAT3 signaling and interfered with AAA formation induced by AngII in ApoE^–/–^ mice, which indicates a novel potential strategy for the prevention and therapy of AAA.

## Introduction

Abdominal aortic aneurysm (AAA) is a potentially life-threatening degenerative vascular disease affecting 6% to 9% of men over the age of 65 years, with an annual death toll of more than 15,000 (Baxter et al., 2008; Wang et al., 2013; Argyriou et al., 2018). Although in most patients no symptoms manifest, AAA progresses over time and eventually ruptures, leading to a high mortality rate (Hoornweg et al., 2008; Ohno et al., 2018). Currently, there is no pharmaceutical strategy that diminishes aneurysm progression during the early stages. Surgery and endovascular repair with stents are the main treatments for AAA (Wang et al., 2013; Ijaz et al., 2016). There is an existing gap in the study of the occurrence and development of AAA, suggesting that exploring potential mechanisms could play a critical role in the prevention and treatment of abdominal aortic aneurysm in clinical work.

The formation of AAA is a complex process, involving remodeling of the extracellular matrix (ECM), chronic inflammation and the degradation of elastin fibers regulated by matrix metalloproteinases (Nordon et al., 2011). A number of reports have demonstrated an increased expression of matrix metalloproteinases (MMPs) in AAA and genetic variants have been proposed to be associated with AAA (Dilme et al., 2014). MMPs, particularly MMP2 and MMP9, degrade the extracellular matrix and elastic fibers leading to the development and progression of AAA (Dilme et al., 2014; Ghosh et al., 2015).

Recently, aortic wall inflammation, which is considered to be the most significant causative factor contributing to the degradation and remodeling of the ECM, has been highlighted in the development and progression of AAA (Ohno et al., 2018). Interleukin-6 (IL-6), one of the proinflammatory cytokines, exerts its effect *via* the IL-6 receptors (IL-6R) and induces homodimerization with its co-receptor gp130, resulting in the phosphorylation of the transcription factor STAT3 (Ferreira et al., 2013). It has been reported that IL-6 signaling – including the expression of IL-6 and phosphorylation of STAT3 (P-STAT3) – is over-activated in AAA lesions (Liao et al., 2012). Genetic studies have shown an association between genetic variation in IL-6R and the risk of developing AAA (Harrison et al., 2013), indicating that targeting IL-6R may be a useful strategy in combatting AAA. These studies suggest that the IL-6/GP130/STAT3 signaling pathway may play an important role in the formation and development of AAA. Inhibition of the IL-6/GP130 interface, and hence influencing the phosphorylation of STAT3, may be a new therapeutic option for AAA.

Bazedoxifene (BAZ) has been approved by the FDA (Food and Drug Administration) for the prevention and treatment of postmenopausal osteoporosis. In our previous study, using multiple ligand simultaneous docking (MLSD) and drug repositioning approaches, we identified that BAZ exhibited a new function targeting the IL-6/GP130 protein-protein interface (Li et al., 2014). BAZ could suppress tumor growth and induce apoptosis in human cancer cells and in a tumor xenograft mice model (Li et al., 2014; Chen et al., 2018). Whether BAZ is effective at suppressing IL-6/GP130/STAT3 signaling or inhibiting the formation of AAA is still unclear. Herein, we reported the suppressive effect of BAZ on the formation and development of AAA. We found that BAZ attenuated the development and severity of AngII-stimulated AAA in ApoE^−/−^ mice and that BAZ could suppress the phosphorylation of STAT3 and the expression of MMP2 and MMP9. Moreover, a similar effect of BAZ was shown in mouse vascular smooth muscle cells (VSMCs). These results may indicate that BAZ exhibits inhibition against the IL-6/GP130/STAT3 signaling pathway and may be promising for use in the prevention or treatment of AAA patients in future.

## Materials and Methods

### Animal Experiment

All animal experiments were carried out in accordance with National Institute of Health guidelines and approved by the Experimental Animal Research Committee of Tongji Medical College, Huazhong University of Science and Technology. Mice were anesthetized using 2% isoflurane mixed with 0.5-1.0 L/min 100% O_2_. We used a classic AAA model in which a continuous AngII infusion in 8-week-old male apolipoprotein-E-deficient (ApoE^−/−^) mice induces AAA formation after implantation by subcutaneously implanted mini-osmotic pumps (Model 2004, Alzet, CA, USA) (Vorkapic et al., 2016). All ApoE^−/−^mice were randomly divided into three groups: control (n=12), AngII (n=13), BAZ (n=12). AngII powder (Sigma) was solubilized in 0.9% sodium chloride and loaded into mini-osmotic pumps for systemic hormone delivery (1000 ng/kg/min infusion rate and 28-day duration) following subcutaneous implantation in the dorsum of mice. ApoE^−/−^ mice in the control group were infused with 0.9% NaCl. The AngII-infused mice were then randomized into two groups (both were fed a normal diet), one group was treated with a vehicle control and the other was given 5mg/kg BAZ (purchased from Cayman Chemical Company, Ann Arbor, Michigan, USA) every day during Ang II infusion. BAZ was dissolved in a PBS solution containing 20% hydroxypropyl-beta-cyclodextrin (HPBCD) and 5% DMSO. After 28 days, aorta tissues were harvested from euthanized mice.

### Histology

The aortas were embedded in paraffin and cut into 5–10μm cross-sections, then stained with hematoxylin and eosin (H&E), or elastica van Gieson (EVG) staining for elastin.

### Immunohistochemistry

The tissue sections were deparaffinized and dehydrated by fractionation using xylene and ethanol. The sections were incubated for 1h at room temperature or overnight at 4°C with the following primary antibodies: IL-6 (anti-rabbit, D220828, Sangon, Shanghai, China), P-STAT3 (Tyrosine 705, #9145, CST), MMP2 (sc-8835, Santa Cruz), MMP9 (sc-6841, Santa Cruz), α-smooth muscle actin (α-SMA, Clone 1A4, DAKO) and CD68 (#76437, CST). After washing with PBS, tissue sections were incubated with biotinylated secondary antibody for 90 min at room temperature. Then an avidin-biotin peroxidase complex was added for another 30 minutes. Then diaminobenzidine was added as a substrate that reacts with immune cells to make stain and then the tissue sections counterstained with hematoxylin.

### Cell Culture and Treatment

The mouse vascular smooth muscle cell (VSMC) line was purchased from American Type Culture Collection. All cells were cultured in a humidified 37°C incubator grown with 5% CO_2_, using DMEM (Dulbecco’s modified Eagle’s medium) containing 10% fetal bovine serum (FBS, Gibico) and 1% penicillin/streptomycin (Sigma).

### Western Blot Analysis

After pretreatment with different concentrations of BAZ (10, 15, 20 μmol/L) or DMSO for 2h, VSMCs were induced by IL-6 or AngII for 30 minutes or 12h before collection. The collected cells lysed in a modified RIPA buffer containing phosphatase inhibitors and protease inhibitors. The protein concentration was detected by a BCA protein assay kit. Proteins in each sample were subjected to SDS-PAGE, then the following primary antibodies were used for western blotting: MMP2 (sc-8835, Santa Cruz), P-STAT3 (Tyrosine 705, #9131, CST), MMP9 (sc-6841, Santa Cruz), and GAPDH (#2118, CST). Blots were developed with horseradish peroxidase-conjugated conjugated secondary antibodies and protein detection was performed using an enhanced chemiluminescence (ECL) western blot kit according to the manufacturer’s instructions.

### ELISA

Vascular smooth muscle cell lines were seeded in 48-well plates (3×10^4^ cells/well) and starved for 10h. Cells were treated with AngII (10^-7^mol/L) or DMSO for 0h, 2h, 4h, 8h, 12h or 24h. Then cell culture medium was collected for ELISA (Rat intact PTH ELISA Kit, Elabscience, Wuhan, China).

### Colony Formation Assay

VSMCs were plated with 1×10^3^ cells/well in 6-well plates then BAZ (15, 20 μM) or DMSO was added for 4 h. After treatment 5000 live cells were reseeded on 10 cm plates with no-drug medium and incubated for 14 days. Colonies were then washed with PBS for three times and stained with crystal violet (0.5%), after fixing with paraformaldehyde (4%) for 20 min. After the crystal violet was removed, plates were washed with PBS and dried.

### Wound Healing

Approximately 2×10^5^ cells were seeded in 6-well plates. A linear scratch was generated with a 10-µl pipette tip after the cells reached 100% conﬂuence and were then washed with PBS to remove non-adherent cells. After treatment with 10μmol/L or 15μmol/L BAZ or DMSO for 2 hours, the medium was changed and fresh medium containing 10% FBS was added. The distance migrated was observed after 12 h or 24 h under the microscope. The cell migration ratio was calculated as following: Cell migration ratio (%) = (specific day wound surface area − initial wound area)/initial wound area × 100%.

### Immunoﬂuorescence Staining Analysis

VSMCs cells were seeded into 6-well plates with pre-placed sides. After the cells are attached, the cells were pretreated with BAZ for 2h, then stimulated by IL-6 for 30 mins. After processing, the cells were washed and then fixed with paraformaldehyde for 15 minutes at room temperature. After two washes with PBS, a PBS buffer containing 0.3% Triton X-100 and 5% normal goat serum was added to permeabilize cells at room temperature for one hour. Next the cells were incubated with a polyclonal rabbit antibody P-STAT3 (1:50 dilution) or STAT3 (1:100 dilution) overnight at 4°C. The cells were washed with a PBS buffer supplemented with 0.1% Tween-20 three times after the overnight incubation. Subsequently, the cells were incubated with Cy3-conjugated anti-rabbit secondary antibody (1:100; Jackson ImmunoResearch Laboratories, West Grove, PA) at room temperature for 1 h and then stained with nuclear−specific DAPI (vector Laboratories, Burlingame, CA, USA) for 5 min. Digital images were captured by ﬂuorescent microscopy.

### Quantitative PCR Analysis

VSMCs were treated with BAZ (20 μmol/L) or DMSO for 2 hours. Then AngII or IL-6 was added for 30 minutes or 4 hours before cells were collected. Total RNA was extracted with an HI Pure RNA extract Kit (Magen, China) and converted to cDNA using a Rever Tra Ace qPCR RT kit (TOYOBO, Japan). The silencing efficiency was detected by RT-PCR performed on the ABI Step One Plus (Applied Biosystems, USA) with SYBR green PCR mix (TOYOBO, Japan) according to the manufacturer’s instructions. The specific oligos used in the study were as follows: MMP2 (forward): 5′-ACACCAAGAACTTCCGACTATCCAATG-3′, MMP2 (reverse): 5′-CAGTACCAGTGTCAGTATCAGCATCAG -3′; MMP9(forward): 5′-CTCCTGGTGCTCCTGGCTCTAG-3′, MMP9(reverse): 5′-GTGTAACCATAGCGGTACAGGTAATCC-3′, GAPDH (forward): 5′-AGTGCCAGCCTCGTCTCATA-3′, GAPDH (reverse): **5-′**AGAGAAGGCAGCCCTGGTAA-3′. The fold change of relative mRNA expression was calculated using the 2-ΔΔCt method.

### Cell Viability Assay

Cell Counting Kit-8 (CCK-8) assay kits (Promoter Biotechnology Ltd, Nanjing, China) were used to detect the cell viability. Cells were planted in a 96-well plate with 1×10^4^ cells/well. After attachment, VSMCs were treated with different dosages of BAZ (5, 10, 15, 20 µM) or DMSO for 24 h. CCK‐8 was added (10 μL/well), and the absorbance was measured at a wavelength of 450 nm.

### Statistical Analysis

Data were presented as the mean ± SEM. The difference between groups was evaluated by one-way ANOVA (Analysis of variance) with Bonferroni’s *post hoc*. Statistical significance was defined as P < 0.05 or P < 0.01. Statistical analysis was performed using SPSS software (version 13.0). All experiments were performed at least three times independently.

## Results

### BAZ Attenuates the Formation and Severity of Aneurysms of AngII-Induced AAA in ApoE^−/−^ Mice

**Figure 1:**
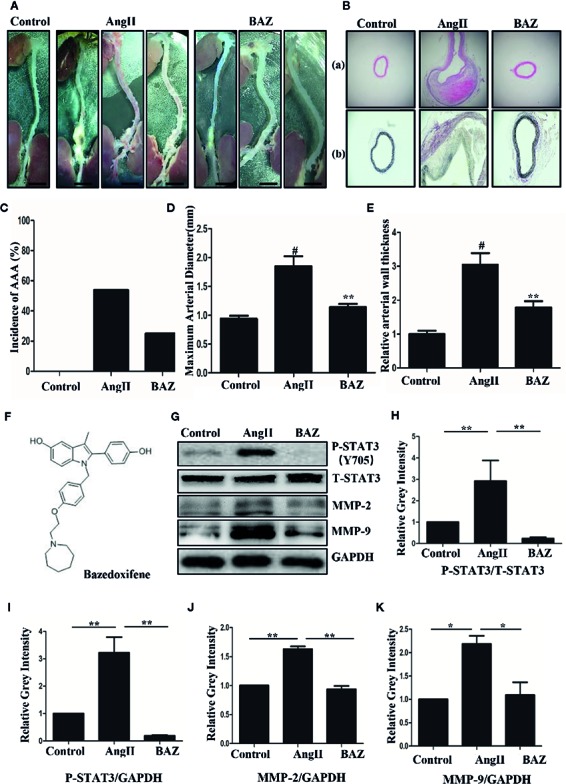
The structure of BAZ and the effect of BAZ on the development of Ang-II-induced abdominal aortic aneurysm **(A)** Morphometrical change of AAA in ApoE^–/–^ mice. Representative images of macroscopic features in aneurysms from sacrificed mice after 28 days. Scale bar denotes 2 mm. **(B)** Histological change of aneurysms in mice shown by hematoxylin eosin (H&E) **(Ba)** and elastin van Gieson (EVG) **(Bb)** staining. Scale bar denotes 400 mm. **(C)** The incidence of AAA was calculated from all of the animals in each group. **(D)** BAZ treatment significantly deceased the external diameter of the aorta (n=9). **(E)** Relative aortic wall thickness of each group (n=9). **(F)** The structure of Bazedoxifene (BAZ). **(G)** Western blots for the expression of P-STAT3, T-STAT3, MMP2 and MMP9 in mice aneurysms. **(H)** Relative gray intensity to STAT3 and **(I–K)** relative gray intensity to GAPDH was calculated (n = 3). Data were expressed as mean ± standard error of the mean. ^#^P < 0.05 compared with the control group, *p < 0.05, **p < 0.01 compared with the AngII group.

We detected the suppressive effect of BAZ in the development of abdominal aortic aneurysm in AngII-induced mice. As shown in **Figure 1A**, compared to the AngII-induced group, BAZ significantly attenuated the formation and severity of abdominal aortic aneurysm. An aortic diameter increase of approximately 50% was defined as the development of AAA (Tsai et al., 2013). As shown in **Figure 1C**, the incidence of AAA was calculated from all the animals in each group. Continuous infusion of AngII in mice increased the incidence of AAA (7/13, 53.84%) compared with the control group (0/12, 0%). However, the incidence of AAA significantly decreased with BAZ treatment (3/12, 25%) in comparison to the AngII group (7/13, 53.84%). Moreover, the external diameter of the aorta was also attenuated by treatment with BAZ when compared with AngII-induced group in APOE^–/–^, as shown in **Figure 1D**. During the intervention period of 28 days, there were four deaths (4/13, 30.77%) in the AngII group, three deaths (3/12, 25%) in the BAZ group and no deaths or aneurysms were observed in the control group (**Supplementary Figure 1**). The survival curves were analyzed with Logrank test. The mortality rate of AngII-induced mice was slightly decreased by treatment with BAZ, when compared to the AngII group at the end of the experiment (day 28). Although there was no significant difference between the AngII and BAZ groups (P=0.23). These results indicated that treatment with BAZ attenuated the formation of AAA in Ang II-induced mice and decreased the expansion of the aorta. The structure of BAZ was shown in **Figure 1F**.

The part of the aortas with maximum diameter were collected and embedded. H&E staining of cross-sections showed that AngII infusion led to a significant thickening of the abdominal aortic wall. While BAZ treatment markedly reduced wall thicknesses (**Figure1Ba**). The relative thickness of the aortic wall was further determined. As shown in **Figure1E**, Baz treatment significantly decreased the aortic wall thickness compared with AngII. In addition, BAZ treatment significantly reduced elastin degradation in AAA lesions of AngII-infused ApoE^−/−^ mice. EVG stained sections suggested that elastin fibers exhibited apparent discontinuity and disintegration in the aortic wall of the AngII-infused mice. While treatment with BAZ obviously reversed the degradation of elastin induced by AngII (**Figure 1Bb**). These results indicated that BAZ could significantly attenuate the development and severity of aneurysms in AngII-infused ApoE^−/−^ mice.

### BAZ Inhibits Expression of P-STAT3, MMPs and Attenuates Aortic Wall Remodeling and Inflammation in AngII Treated ApoE^−/−^ Mice

**Figure 2:**
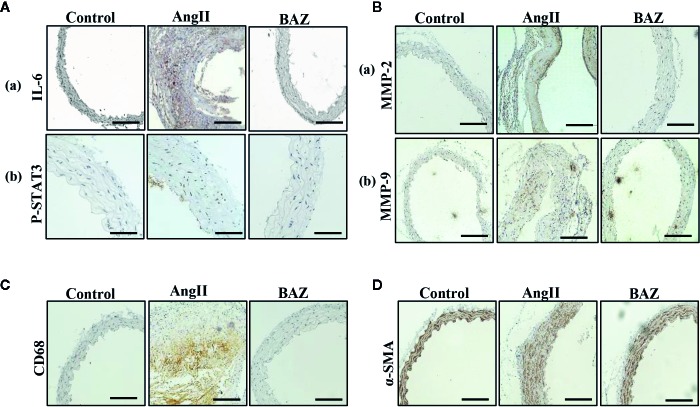
Effect of BAZ on the abdominal aorta *via* immunohistochemical staining in mice. **(A)** Expression and distribution of P-STAT3 and IL-6 (both brown-stained) in cross-sections of aorta from ApoE^–/–^mice. **(B)** The expression of MMP2 and MMP9 was detected *via* immunohistochemical staining in the abdominal aorta of ApoE^–/–^ mice. **(C)** Representative immunohistochemical staining of CD68 in aortic cross-sections. **(D)** Representative images showing a-SMA expression in aortic cross-sections. The scale depicts 100μm for each interval in **A(b)** and 200μm in **A(a) B, C, D**.

It has been reported that AngII-infused ApoE knock-out mice mostly present AAAs around the suprarenal area immediately distal to the branch of the renal artery (Daugherty et al., 2006). So, we selected aortic sections from the aneurysm-prone areas for histological characteristics analysis. Representative immunohistochemical staining was shown in **Figure 2**. To detect the effect of BAZ on IL-6/GP130/STAT3 signaling, the expression of P-STAT3 in abdominal aortas of three groups of mice was compared. Western blot analysis showed that AngII infusion led to increased phosphorylation of STAT3 at Tyr705, however, BAZ could inhibit the expression of P-STAT3 (**Figures 1G–I**). Immunohistochemistry showed similar results. As in **Figure 2Ab**, the expression of P-STAT3 was increased (brown nuclei represent P-STAT3 positive) in the vascular wall of AngII-induced mice. BAZ could decrease the expression of P-STAT3. As shown in **Figure 2Aa**, the level of IL-6 was increased in the vessel wall of abdominal aortic aneurysms in AngII-infused ApoE^-/-^ mice compared to similar sections of aorta in saline-infused control mice. While treatment with BAZ could significantly decrease the expression of IL-6 in the same vessel wall section of aortas. Simultaneously, we also tested the expression of IL-6 in mouse serum *via* ELISA. As shown in **Supplementary Figure 2**, the expression of IL-6 in serum was increased dramatically in Ang II-induced mice. However, BAZ treatment deceased the expression of IL-6 in serum compared with only AngII-infused mice. MMPs, especially MMP2 and MMP9, whose expressions were increased in the abdominal aortas of AngII-induced ApoE^−/−^ mice, participate in the process of AAA development. Immunohistochemical staining and western blotting showed that administration of BAZ significantly down-regulated the expression of MMP2 and MMP9 (**Figures 1G, J, K**, **2B**). Abundant infiltration by CD68-positive macrophages was detected in the adventitia and media of the aortic aneurysms from the AngII-infused mice. Increased expression of CD68 was detected in the suprarenal section of aortas in AngII-induced group without BAZ, while the expression of CD68 was significantly inhibited by BAZ (**Figure 2C**). Hyperproliferation of VSMCs in the aortic wall plays an important role in AAA. Our results indicated that BAZ inhibited the over-proliferation of VSMCs in AAA lesions of AngII-infused ApoE^–/–^ mice. As shown in **Figure 2D**, treatment with BAZ decreased the expression of α-SMA when compared with AngII-infused mice.

### Bazedoxifene Inhibits the Phosphorylation of STAT3 Induced by IL-6

**Figure 3:**
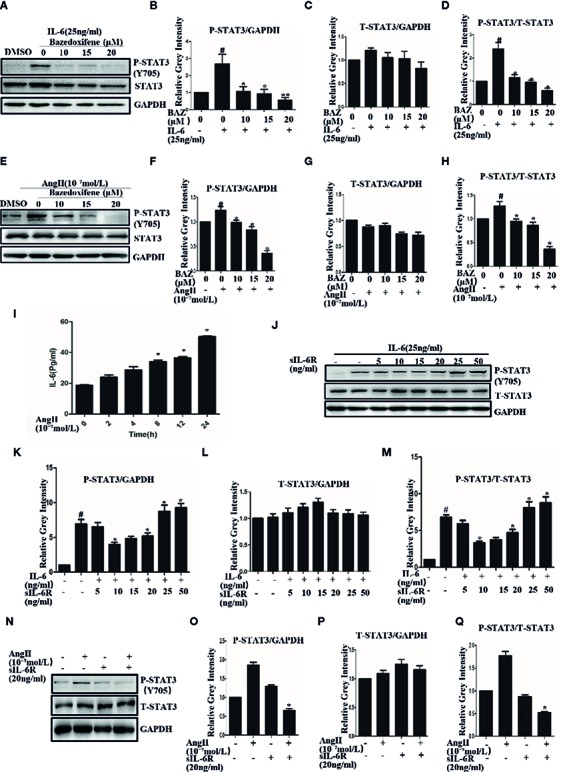
BAZ suppresses the phosphorylation of STAT3 induced by IL-6 and AngII in VSMCs. Cells were pretreated with BAZ for 2 hours and then stimulated with IL-6 **(A)** and AngII **(E)**, western blotting was used to show the expression of P-STAT3 and STAT3. **(I)** ELISA was used to exam the effect of AngII on the secretion of IL-6 *in vitro*. **(J)** The effect of sIL-6R at different concentrations on IL-6 signaling transduction. **(N)** The effect of AngII or sIL-6R on the expression of P-STAT3. **(B, C, F, G, K, L, O, P)** Relative gray intensity to GAPDH was calculated. **(D, H, M, Q)** Relative gray intensity to T–STAT3 was calculated. Data were expressed as mean ± standard error of the mean (n = 3). ^#^P < 0.05 compared with the control; *P< 0.05, **P< 0.01compared with IL-6 or AngII.

Vascular smooth muscle cells, which act as a major component of the aortic wall, play an important role in AAA (Yao et al., 2019). VSMCs were used to test the effect of BAZ *in vitro*. Our results showed that Interleukin-6 could induce the phosphorylation of STAT3 in VSMCs (**Figures 3A, B**). BAZ inhibited the phosphorylation of STAT3 induced by IL-6 but had no significant effect on the overall expression of STAT3 in VSMCs (**Figures 3A–D**).

### Bazedoxifene Suppresses the Phosphorylation of STAT3 Induced by AngII

We also found that AngII could induce phosphorylation of STAT3 in VSMCs (**Figures 3E, F**). Similarly, BAZ, which has been reported to inhibit the IL-6/GP130 interface, could also suppress the phosphorylation of STAT3 as induced by AngII and without an effect on overall STAT3 levels (**Figures 3E–H**).

To explore the mechanism, we first detected the effect of AngII on IL-6 secretion. When the cells were stimulated with AngII, the concentration of IL-6 was increased in the supernatant. This result suggested that AngII could induce the secretion of IL-6 in VSMCs (**Figure 3I**). It has been reported that in the classic IL-6 signaling pathway IL-6 binds to the membrane-bound IL-6 receptor (IL-6R) and then recruits GP130 to form the IL-6/IL-6Rα/GP130 heterotrimer which is followed by the activation and phosphorylation of STAT3. However, in IL-6 trans signaling IL-6 can also bind to soluble forms of IL-6R (sIL-6R) and then bind to gp130 (Ferreira et al., 2013; Aparicio-Siegmund et al., 2019). So we further detected the effects of sIL-6R on IL-6 or Ang II induced STAT3 phosphorylation. As shown in our results, sIL-6R could slightly decrease IL-6-induced STAT3 phosphorylation at lower concentrations but increased STAT3 phosphorylation at higher concentrations (**Figures 3J–L**). It was very interesting to find that the phosphorylation of STAT3 induced by AngII could be inhibited by sIL-6R (**Figures 3N–P**). The density of pSTAT3 was normalized by the total density of STAT3 (**Figures 3D, H, M, Q**). These data indicated that AngII-induced activation of STAT3 was at least partially mediated by the IL-6 signaling pathway.

### BAZ Suppresses the Expression of MMP2, MMP9 Induced by IL-6 and AngII

**Figure 4:**
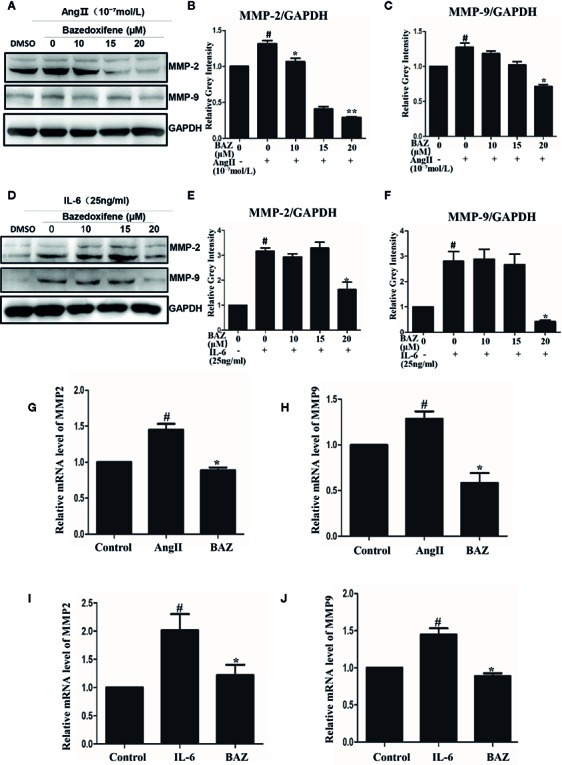
BAZ suppresses the expression of MMP2 and MMP9 induced by IL-6 and AngII in VSMCs. Pretreatment of BAZ for 2 hours suppressed the increase in both MMP2 and MMP9 expression induced by IL-6 **(A)** and AngII **(D)**. Relative gray intensity to GAPDH was calculated (n = 3) **(B, C, E, F)**. The mRNA expression of MMP2 and MMP9 was assessed by qRT-PCR and normalized to GAPDH and is expressed in arbitrary units. BAZ suppressed the mRNA expression of MMP2 and MMP9 induced by IL-6 **(I, J)** and AngII **(G, H)**. Data were presented as the mean ± SEM. ^#^P < 0.05 compared with the control; *P< 0.05, **P< 0.01 compared with IL-6 or AngII.

In our results, western blotting showed that BAZ suppressed the expression of MMP2 and MMP9 induced by IL-6 and AngII (**Figures 4A, D**). Relative gray intensity to GAPDH was shown in **Figures 4B-F**. Moreover, we found the mRNA expression of MMP2 and MMP9 induced by IL-6 and AngII was decreased by BAZ (**Figures 4G–J**).

### BAZ Inhibits STAT3 Activation in the Nucleus in VSMCs

**Figure 5:**
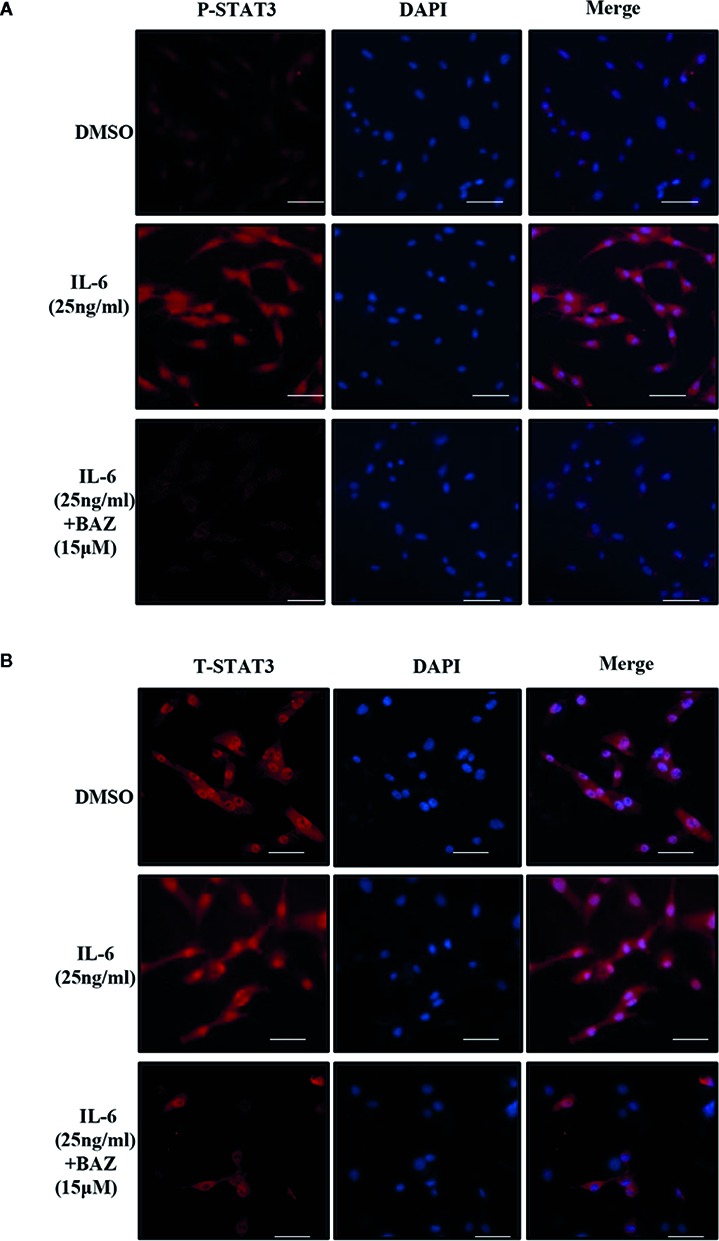
BAZ suppresses STAT3 activation in nucleus and the nuclear translocation induced by IL-6 in VSMCs. After pretreatment with BAZ for 2 hours, cells were stimulated with IL-6 for another 30 minutes, and then STAT3 activation **(A)** and STAT3 nuclear translocation **(B)** were detected by immunofluorescence staining as described. Scale bar represents 200μm.

After pretreatment with BAZ for 2 hours, VSMCs were induced with IL-6 for another 30 minutes and P-STAT3 or STAT3 was detected by immunoﬂuorescence staining. After stimulation by IL-6, STAT3 was phosphorylated and translocated into the nucleus in VSMCs. However, most of the STAT3 was retained in the cytoplasm of cells treated with BAZ (**Figure 5B**). In addition, IL-6 induced STAT3 phosphorylation in the nucleus, which was blocked by BAZ in VSMCs (**Figure 5A**). Thus, these data suggested that the suppression of STAT3 phosphorylation by BAZ might impair STAT3 transcriptional functions in VSMCs.

### BAZ Suppresses Cell Migration, Colony Forming Capacity and Cell Viability in VSMCs

**Figure 6:**
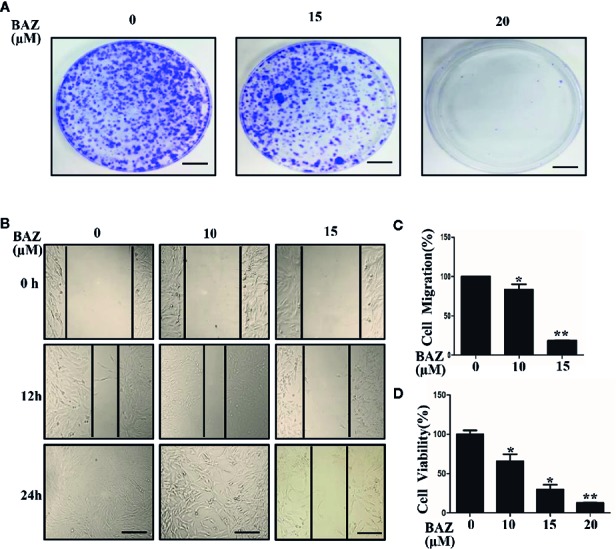
BAZ inhibits colony formation and wound healing in VSMCs. **(A)** Cells were pretreated with BAZ (15 or 20 mM) or DMSO for 4 h, then colony formation was evaluated after two weeks. Scale bar denotes 1 cm. **(B)** After BAZ treatment for 2h, the migration ability of VSMCs was detected using wound-healing assays. Scale bar represents 400 μm. **(C)** Cell migration was assessed. **(D)** BAZ reduced cell viability of VSMCs. *P< 0.05, **P< 0.01 compared with the control.

STAT3 phosphorylation was involved in the over-activation of vascular smooth muscle cells. We next evaluated whether BAZ could inhibit cell migration and colony formation, which are important processes in the development of abdominal aortic aneurysms. As shown in **Figure 6A**, VSMCs were pretreated with BAZ for 4 h, then the same number of viable cells were planted at the same cell densities in 10 cm plates. After incubation for two weeks, cells were stained with crystal violet. BAZ could clearly inhibit colony formation in VSMCs. Wound healing assays were used to detect the effect of BAZ on cell migration in vascular smooth muscle cells. Our results showed that treatment with BAZ caused a reduction in wound healing (**Figures 6B, C**). Meanwhile, the CCK-8 assay suggested that BAZ suppressed cell viability in VSMCs (**Figure 6D**). These results may indicate BAZ could suppress the formation of AAA *via* the inhibitory effect on over-proliferation in VSMCs.

## Discussion

Abdominal aortic aneurysm is a serious vascular disease with a high mortality rate and no effective therapeutic treatment is available except for aneurysmectomy (Erbel et al., 2015). Despite the fact that animal studies have identified several potential therapeutic targets in the pathogenesis of AAA, pharmacotherapy for AAA is yet to be established (Baxter et al., 2008; Ohno et al., 2018). It has been reported that there was no significant association between AAA progression and the use of statins, beta blockers, angiotensin-converting enzyme inhibitors or angiotensin II receptor blockers (Baxter et al., 2008; Kokje et al., 2015), which indicated that there may be other mechanisms involved in the progression of AAA. In our results, BAZ could inhibit the activity of the IL-6/GP130/STAT3 signaling pathway and significantly decreased the severity of AAA in an AngII-induced ApoE^−/−^ mice model. BAZ could also inhibit the expression of P-STAT3, MMP2 and MMP9 *in vivo* and *in vitro*. It could also inhibit the proliferation of VSMCs and maladaptive responses to inflammation which accompanied the overexpression of pro-inflammatory mediators *in vivo* (Butoi et al., 2016; Das et al., 2017). These results suggest that BAZ may have a protective effect on the treatment of AAA by inhibition of the IL-6/GP130/STAT3 signaling pathway. That may provide a new option for the prevention and treatment of AAA.

Although the pathogenesis of AAA remains unclear, a large body of evidence supports the critical role of inflammation (Aoki et al., 2007). IL-6 as a multicellular cytokine has been reported to participate in cell proliferation, apoptosis and various inflammatory responses (Hunter and Jones, 2015). In an inflammatory response, IL-6 exerts its function through binding to its receptor (IL-6R, located on cell membranes, or stays in a soluble state in plasma) by forming a binary complex and then recruiting glycoprotein 130 (GP130) leading to the formation of the IL-6/IL-6R/GP130 heterotrimer. The homodimerization of the IL-6/IL-6Rα/GP130 trimers initiate an intracellular signaling cascade of phosphorylation of Janus kinases (JAKs), thereby activating a downstream effector STAT3 *via* phosphorylation (Li et al., 2014). Genetic and observational associations imply a link between IL-6 and AAA disease (Hunter and Jones, 2015). Furthermore, a systematic review and meta-analysis suggested that the IL-6 receptor pathway might be a causal signaling in human AAA pathogenesis and inhibition of IL-6R may contribute to AAA treatment (Harrison et al., 2013). STAT3, one of the significant downstream target genes of IL-6, has been documented in the pathogenesis of AAA (Ohno et al., 2018; Yao et al., 2019). It has been reported that an increased expression of P-STAT3 is identified in AAA tissues compared with non-aneurysmal controls (Liao et al., 2012). Moreover, in our results, BAZ, which has been reported to inhibit the IL-6/GP130 protein-protein interface, significantly decreased the severity of AAA in ApoE^−/−^ mice. Furthermore, BAZ could suppress the phosphorylation of STAT3 *in vitro* and *in vivo*. These results may suggest that the IL-6/GP130/STAT3 pathway may take a significant role in the pathogenesis of abdominal aortic aneurysm. More importantly, the inhibition of the IL-6/GP130/STAT3 pathway might become a new target for the prevention or treatment of AAA.

A number of studies have shown that AngII induces AAA by increasing the inflammatory profile, however, its specific molecular pathway remains unclear (Weiss et al., 2014). Despite the fact that animal studies have shown that the use of AT1 receptor inhibitors may protect against abdominal aortic aneurysms, the outcomes of clinical research has been unsatisfying and often remains inconsistent (Maekawa et al., 2017). Use of AT1-receptor antagonists does not affect AAA growth (Weiss et al., 2014). It indicates that there may be other molecular pathways involved in the occurrence and development of abdominal aortic aneurysms induced by AngII. However, our results demonstrated that AngII-induced activation of STAT3 was mediated by IL-6, at least in part. We found P-STAT3 induced by AngII could be inhibited by soluble IL-6 receptors (sIL-6R), a specific constituent of the trans-signaling of IL-6, which could limit IL-6-induced P-STAT3 at low concentrations but increase P-STAT3 at higher concentrations. Furthermore, BAZ, which could impede the IL-6/GP130 interface, could also inhibit the phosphorylation of STAT3 induced by AngII. These results indicate that IL-6/GP130/STAT3 may participate, albeit partially, in the response to AngII in VSMCs. This may provide a theoretical basis for the treatment of AAA by targeting the IL-6/GP130/STAT3 signaling pathway.

Bazedoxifene has been approved by the FDA for use as a treatment for osteoporosis in clinics. It has been reported that BAZ could reduce cerebral aneurysm ruptures in rats in a blood pressure-independent manner and that BAZ has no significant effect on blood pressure (Maekawa et al., 2017). Moreover, in our previous research, we demonstrated that Bazedoxifene could bind to the GP130 D1 domain and inhibit the IL-6/GP130 protein-protein interface (Li et al., 2014). Subsequently, it suppresses STAT3 phosphorylation and transcription induced by IL6. Various studies have shown Bazedoxifene could inhibit tumor growth by targeting the IL-6/GP130/STAT3 signaling pathway (Wu et al., 2016; Xiao et al., 2017; Chen et al., 2019; Ma et al., 2019). Based on our study, we found Bazedoxifene could attenuate the formation and severity of aneurysms, which may expand the application of Bazedoxifene to the prevention and treatment of abdominal aortic aneurysms in clinics. Despite reports that some small molecule compounds, such as Ursolic acid (Zhai et al., 2018) and S31-201 (Qin et al., 2015), could decrease the incidence and severity of AAAs by inhibiting the phosphorylation of STAT3 in an AngII-infused ApoE^–/–^ mouse model, to date no small molecule STAT3 inhibitor is available for clinical therapy. Compared with these compounds (which are not used clinically), Bazedoxifene has advantages in terms of stability, security and oral absorbency. Drug repurposing is a valuable approach in delivering new AAA therapeutics rapidly into clinics.

The limitations of our study require consideration. Several animal models of AAA have been developed in mice including injury of the aortic wall with calcium chloride or elastase (Patelis et al., 2017). Compared with other models, the AngII-infused model supports an imbalance of the renin-angiotensin system in the pathogenesis of AAA (Zhang et al., 2009). Infusion of AngII *via* subcutaneous osmotic minipumps in ApoE^–/–^ mice has also been shown to result in the formation of AAAs, which could mimic the inflammatory microenvironment and exhibit many characteristics of human AAA including rupture of the elastic layer, activation of matrix metalloproteinases, macrophage infiltration and so on (Senemaud et al., 2017). Differences between species and individuals perhaps lead to diverse consequences. For example, unlike humans, mice often present suprarenal AAAs and their relevance remains somewhat limited by their inability to expand indefinitely with time (Patelis et al., 2017). So further experiments might be needed to evaluate the effect of BAZ in human AAA. On the other hand, although our results suggested a significant effect of BAZ on AAA in VSMCs and in animals, whether the *in vivo* effect of BAZ was dependent on the IL-6/GP130/STAT3 signaling pathway was still unclear in our work due to the complicated mechanisms of AAA.

In summary, we demonstrated that BAZ might play a protective role in the pathology of AAA in AngII-induced mice. As an FDA-approved drug with known pharmacokinetics and safety, Bazedoxifene has great potential to be used in clinics for the treatment of AAA. Moreover, considering BAZ as a pharmacological template, we established a basis for increasing the rate of development of drugs that selectively target the IL-6/GP130/STAT3 pathway with better bioavailability and fewer side effects. It may provide a novel strategy for the prevention and therapy of AAA in clinics.

## Data Availability Statement

All datasets generated for this study are included in the article/**Supplementary Material**.

## Ethics Statement

All animal experiments were carried out in accordance with National Institute of Health guidelines and approved by the Experimental Animal Research Committee of Tongji Medical College, Huazhong University of Science and Technology.

## Author Contributions

CZ and LL contributed to the design of this study, coordinated the experiments and provided funding sources. DY and HM performed animal experiments and biological molecular experiments. WS, PL, TL, and MZ contributed to the cellular experiments. JG, JT, and SH contributed to the collection and analysis of the data. CL and JL provided extensive support. SL and JGL contributed to the manuscript draft. All authors have contributed significantly and approved the final manuscript.

## Funding

This research was supported by the National Natural Science Foundation of China to LL (81570416) and SL (81570337), the Fundamental Research Fund for the Central Universities (HUST) to JGL (2017KFYXJJ099), the Science and Technology Project Foundation of Wuhan to JGL (2017060201010175), Hubei Province health and family planning scientific research project to JGL (WJ2019M120), Natural Science Foundation of Hubei Province to LL (2019CFB668) and the Outstanding Young Investigator Foundation of Tongji Hospital to LL (YXQN009).

## Conflict of Interest

The authors declare that the research was conducted in the absence of any commercial or financial relationships that could be construed as a potential conflict of interest.

## Supplementary Material

Figure S1 Kaplane-Meier curves of the survival of APOE^-/-^. Data represent the Kaplan-Meier curve depicting mouse survival. (n=13 for the AngII group, n=12 for the control group and BAZ group. P=0.23, for AngII vs BAZ, log-rank test).

Figure S2 The expression of IL-6 in mice serum. The expression of IL-6 in mice serum was decreased by BAZ treatment compared with AngII-induced (n=9). Data were expressed as mean ± standard error of the mean. #P < 0.05 compared with the control group, *p < 0.05, compared with the AngII group.
