# Supplementary figures and images for: Bazedoxifene Attenuates Abdominal Aortic Aneurysm Formation via Downregulation of Interleukin-6/Glycoprotein 130/Signal Transducer and Activator of Transcription 3 Signaling Pathway in Apolipoprotein E–Knockout Mice
Source: Front Pharmacol. 2020 Apr 17;11:392. doi: 10.3389/fphar.2020.00392 (PMC7180191; doi:10.3389/fphar.2020.00392)

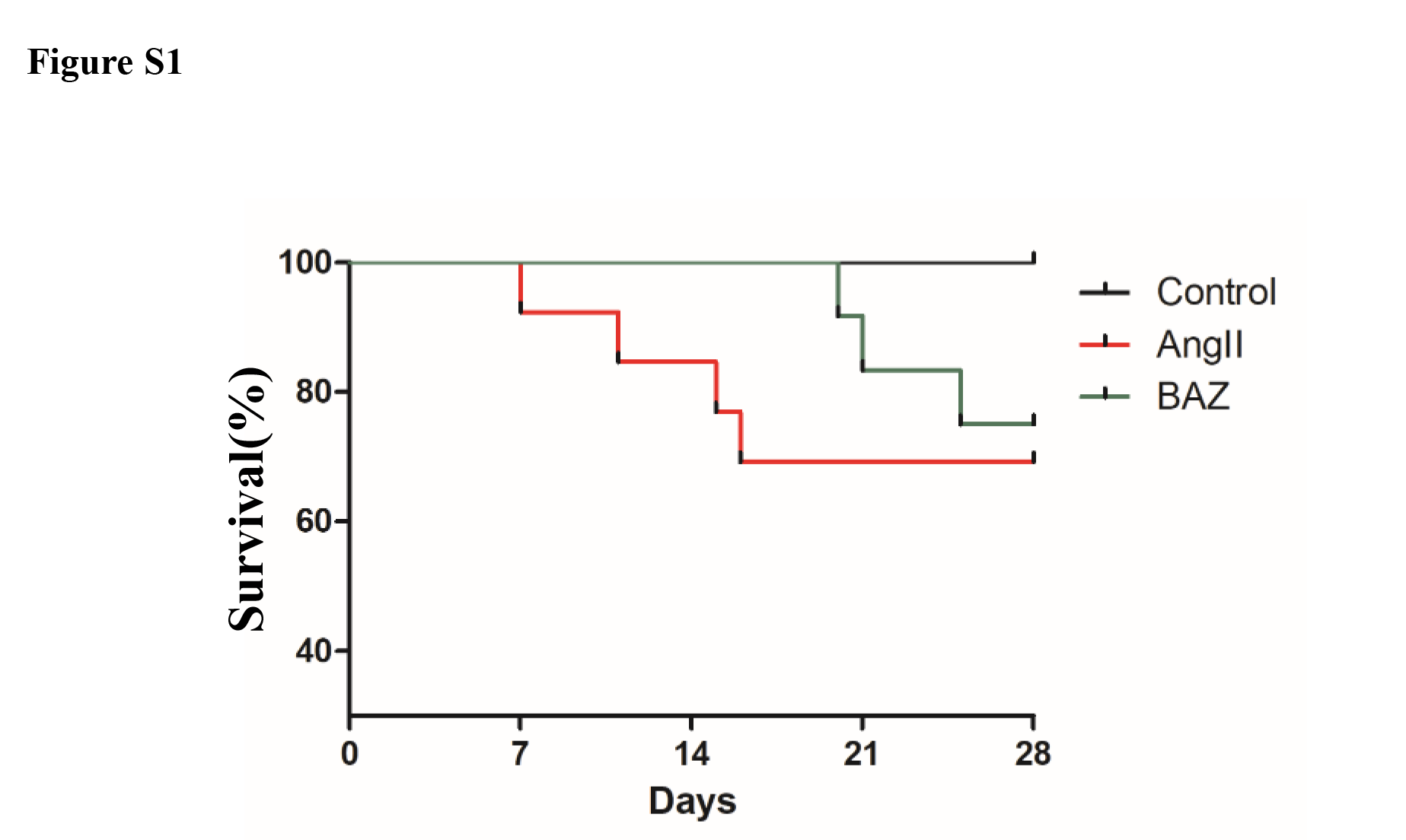

Supplement: Figure S1 — Kaplane-Meier curves of the survival of APOE-/-. Data represent the Kaplan-Meier curve depicting mouse survival. (n=13 for the AngII group, n=12 for the control group and BAZ group. P=0.23, for AngII vs BAZ, log-rank test). [file Image_1.tif]

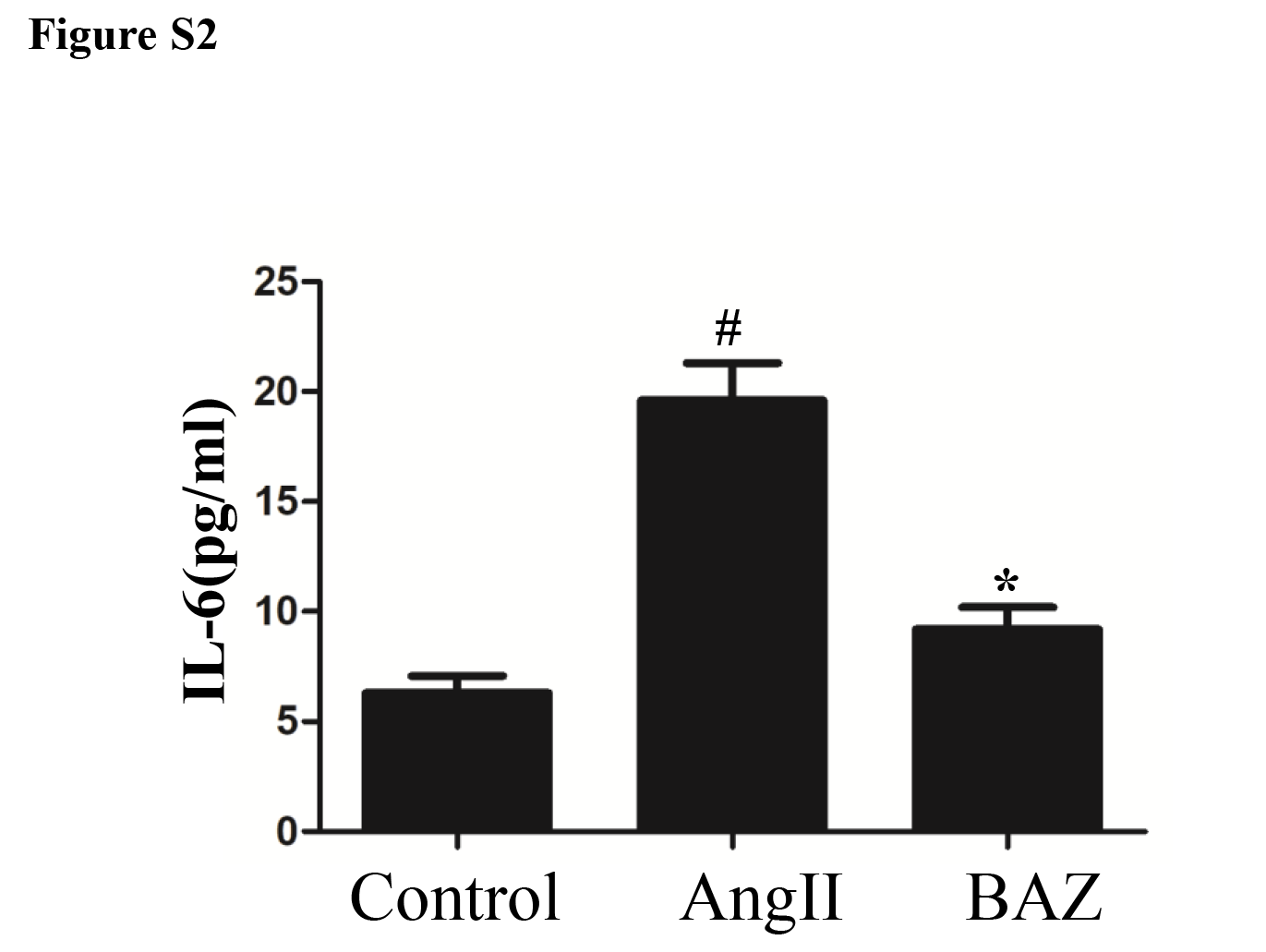

Supplement: Figure S2 — The expression of IL-6 in mice serum. The expression of IL-6 in mice serum was decreased by BAZ treatment compared with AngII-induced (n=9). Data were expressed as mean ± standard error of the mean. #P < 0.05 compared with the control group, *p < 0.05, compared with the AngII group. [file Image_2.tif]
